# Electrical impedance myography detects dystrophin-related muscle changes in *mdx* mice

**DOI:** 10.1186/s13395-023-00331-1

**Published:** 2023-11-18

**Authors:** Tetsuaki Hiyoshi, Fuqiang Zhao, Rina Baba, Takeshi Hirakawa, Ryosuke Kuboki, Kazunori Suzuki, Yoshiro Tomimatsu, Patricio O’Donnell, Steve Han, Neta Zach, Masato Nakashima

**Affiliations:** 1 Neuroscience Translational Medicine, Neuroscience Drug Discovery Unit, Takeda Pharmaceutical Company Limited, 26-1, Muraoka-Higashi 2-Chome, Fujisawa, Kanagawa 251-8555 Japan; 2 Center of Excellence for Imaging, Preclinical and Translational Sciences, Takeda Development Center Americas, Inc., 95 Hayden Avenue, Lexington, MA 02141 USA; 3 Muscular Disease and Neuropathy Unit, Neuroscience Drug Discovery Unit, Takeda Pharmaceutical Company Limited, 26-1, Muraoka-Higashi 2-Chome, Fujisawa, Kanagawa 251-8555 Japan; 4 Neuroscience Translational Medicine, Neuroscience Drug Discovery Unit, Takeda Development Center Americas, Inc., 95 Hayden Avenue, Lexington, MA 02141 USA; 5 Neuroscience Therapeutic Area Unit, Takeda Development Center Americas, Inc., 95 Hayden Avenue, Lexington, MA 02141 USA

**Keywords:** Duchenne muscular dystrophy, Electric impedance myography, Magnetic resonance imaging, *mdx* mice, Cell-penetrating peptide conjugated antisense phosphorodiamidate morpholino oligomer

## Abstract

**Background:**

The lack of functional dystrophin protein in Duchenne muscular dystrophy (DMD) causes chronic skeletal muscle inflammation and degeneration. Therefore, the restoration of functional dystrophin levels is a fundamental approach for DMD therapy. Electrical impedance myography (EIM) is an emerging tool that provides noninvasive monitoring of muscle conditions and has been suggested as a treatment response biomarker in diverse indications. Although magnetic resonance imaging (MRI) of skeletal muscles has become a standard measurement in clinical trials for DMD, EIM offers distinct advantages, such as portability, user-friendliness, and reduced cost, allowing for remote monitoring of disease progression or response to therapy. To investigate the potential of EIM as a biomarker for DMD, we compared longitudinal EIM data with MRI/histopathological data from an X-linked muscular dystrophy (*mdx*) mouse model of DMD. In addition, we investigated whether EIM could detect dystrophin-related changes in muscles using antisense-mediated exon skipping in *mdx* mice.

**Methods:**

The MRI data for muscle T2, the magnetic resonance spectroscopy (MRS) data for fat fraction, and three EIM parameters with histopathology were longitudinally obtained from the hindlimb muscles of wild-type (WT) and *mdx* mice. In the EIM study, a cell-penetrating peptide (Pip9b2) conjugated antisense phosphorodiamidate morpholino oligomer (PPMO), designed to induce exon-skipping and restore functional dystrophin production, was administered intravenously to *mdx* mice.

**Results:**

MRI imaging in *mdx* mice showed higher T2 intensity at 6 weeks of age in hindlimb muscles compared to WT mice, which decreased at ≥ 9 weeks of age. In contrast, EIM reactance began to decline at 12 weeks of age, with peak reduction at 18 weeks of age in *mdx* mice. This decline was associated with myofiber atrophy and connective tissue infiltration in the skeletal muscles. Repeated dosing of PPMO (10 mg/kg, 4 times every 2 weeks) in *mdx* mice led to an increase in muscular dystrophin protein and reversed the decrease in EIM reactance.

**Conclusions:**

These findings suggest that muscle T2 MRI is sensitive to the early inflammatory response associated with dystrophin deficiency, whereas EIM provides a valuable biomarker for the noninvasive monitoring of subsequent changes in skeletal muscle composition. Furthermore, EIM reactance has the potential to monitor dystrophin-deficient muscle abnormalities and their recovery in response to antisense-mediated exon skipping.

**Supplementary Information:**

The online version contains supplementary material available at 10.1186/s13395-023-00331-1.

## Background

Duchenne muscular dystrophy (DMD) is an inherited disorder caused by a recessive mutation in the X chromosome that occurs in approximately 1 in 5000–6000 newborn boys. Patients have mutations in the dystrophin-encoding *DMD* gene, which result in lack of functional dystrophin proteins [1]. Dystrophin deficiency generates a recurring cycle of skeletal muscle fiber degeneration and regeneration, leading to the accumulation of fibrotic and adipose tissues [2]. Cardiac muscle also undergoes degeneration and the accumulation of fibrotic tissues [3]. Owing to muscular abnormalities, most patients become wheelchair-dependent at approximately 10–12 years of age, and most patients with DMD die between 20 and 40 years of age from cardiac and/or respiratory failure [4].

Mice with X-linked muscular dystrophy (*mdx*) lack full-length dystrophin isoforms because of a spontaneous nonsense point mutation in exon 23 of *Dmd* [5]. In preclinical studies, the *mdx* mouse with a C57BL/10 genetic background is commonly used as a mouse model of DMD and is generally recognized as an appropriate model for the evaluation of muscle degeneration and regeneration rather than muscle atrophy [6, 7]. This is because the strain shows intense inflammatory degeneration in muscles from an early age (3–6 weeks) but a relatively mild phenotype overall, due to higher regenerative ability of *mdx* muscle than that in humans [6–9].

In the field of skeletal muscle research, electrical impedance myography (EIM) offers a noninvasive and repetitive measure for quantitatively assessing muscle composition [10, 11]. Unique features of EIM, such as portability, user-friendliness, and low-cost, enable remote monitoring of disease progression and/or therapeutic response, including at patients’ home settings, in several neuromuscular disorders [12–15]. In addition, the application of EIM in clinical trials may reduce sample size and bring cost-related advantages to new drug development [16, 17]. However, the feasibility of using EIM as a translatable biomarker in mouse DMD models is less clear. To assess this, comparative analysis with magnetic resonance imaging (MRI) is important, because MRI is becoming a more widely used biomarker in DMD clinical studies [18]. Although a previous study using aged *mdx* mice (18 months old) demonstrated that EIM reactance and transverse relaxation time (T2) in MRI could discriminate *mdx* mice from wild-type (WT) mice [19], a comparison between EIM and MRI in young *mdx* mice with early disease progression has not yet been conducted. The young *mdx* mice with a dynamic disease progression will help us understand alterations in biomarkers in the disease state and bridge these preclinical results to human studies. From those reasons, young *mdx* mice with acute muscle degeneration and inflammatory pathological features [6–9], which correspond to those seen in the early disease stages of patients with DMD, are suitable for characterizing EIM vs. MRI as a translatable biomarker for DMD.

Regarding potential treatment for DMD, there is growing evidence that the antisense oligonucleotides (ASOs)-mediated exon skipping approach can restore functional dystrophin proteins and improve muscle function, by restoring the open reading frame around the site of dystrophin mutations [20–22]. Preclinical studies have shown that in vivo exon skipping using cell-penetrating peptide-conjugated antisense phosphorodiamidate morpholino oligomers (PMOs) is a powerful strategy [23–26]. Among them, Pip9b2-conjugated PMO (PPMO) exhibited dose-dependent exon skipping and dystrophin protein restoration in the skeletal muscles of *mdx* mice [25]. A potential challenge in turning these treatment approaches into clinical practice is verifying the change in functional dystrophin expression in patients with DMD, which is now achieved only by burdensome muscle biopsy [1]. Thus, if EIM can noninvasively assess dystrophin-related muscle changes, it would be a valuable tool in clinical practice.

In the present study, we investigated the natural history of changes in hindlimb EIM in *mdx* and WT mice and compared them to changes in MRI and muscle histopathology to evaluate the potential usefulness of EIM in the research of neuromuscular disorders. Additionally, we investigated whether EIM could detect dystrophin-related muscle changes in *mdx* mice treated with PPMO.

## Methods

### Animals

Mice were group-housed with access to food and water ad libitum and under a 12 h light/dark cycle (lights on at 7:00 A.M.) at a temperature of 22 ± 1 °C. For MRI study, male *mdx* mice (C57BL/10ScSn-*Dmd*^*mdx*^/J, 001801, *n* = 8) and age-, sex-matched wild-type (WT) mice (C57BL/10ScSnJ, 000476, *n* = 8) were purchased from Jackson Laboratory (Bar Harbor, ME, USA). For EIM studies, male *mdx* mice (C57BL/10ScSn-*Dmd*^*mdx*^/JicJcl, *n* = 43) and WT mice (C57BL/10Sn Slc, *n* = 33) were purchased from CLEA Japan (Tokyo, Japan) and Japan SLC (Hamamatsu, Japan), respectively. For the natural history study of EIM measurements and histopathology, mice were allocated randomly. EIM was continuously recorded from the same individual (*n* = 6 for WT, *n* = 6 for *mdx* mice) at 6, 12, 18, and 24 weeks of age, with muscle tissue sampled for histopathology at 24 weeks of age after all the EIM recordings. Additionally, for histopathology, muscle tissue was sampled after EIM recording at 6, 12, or 18 weeks of age (*n* = 5 or 6/group/time point). For the drug intervention study using EIM, male *mdx* mice (*n* = 20) and WT mice (*n* = 10) at 14–15 weeks of age were used. All experimental procedures were approved by the Institutional Animal Care and Use Committee (IACUC) of Shonan Health Innovation Park or Northeastern University and were conducted in accordance with the guidelines.

### MRI and magnetic resonance spectroscopy (MRS)

These experiments were performed at the Northeastern University (Boston, MA, USA). Mice were initially anesthetized with isoflurane (5% for induction and 2% during setup) and then secured in a prone position on a Bruker cradle. During the study, respiration rate was monitored using a balloon secured under the mouse’s chest and a pressure transducer. Body temperature was measured with a rectal probe and maintained at ~ 36 °C with a feedback-controlled air heater (SA Instruments, Inc., Stony Brook, NY, USA). All MRI measurements were performed using a Bruker Biospec 7.0-T, 20 cm bore USR horizontal system (Bruker Scientific, Billerica, MA, USA). An actively decoupled 2 cm diameter surface coil positioned beneath the thighs/legs of the mouse was used as the radiofrequency (RF) receiver, and an actively decoupled 72 mm diameter volume coil was used as the RF transmitter. Magnetic field homogeneity was optimized using automatic global shimming. Pilot images in three directions (axial, sagittal, and coronal) were acquired using a fast, low-angle shot (FLASH) sequence. A multi-slice multi-echo (MSME) sequence was used for T2 measurement [27–29]. T2 MRI data were acquired from 16 consecutive axial slices with a slice thickness of 1.5 mm, which covered the thighs and legs. The MRI parameters for the MSME sequence were as follows: matrix = 128 × 128, field of view = 25 × 25 mm^2^, in-plane resolution = 0.2 × 0.2 mm^2^, repetition time (TR) = 4 s, first echo time (TE) = 11 ms, 10 echoes with 11 ms echo spacing, no averaging, and total acquisition time = 512 s. Previous studies have reported that factors such as stimulated echoes can cause errors in T2 measurement due to the contiguous slices and/or multiple echoes [30, 31]. The Bruker MSME protocol adopts two mechanisms to minimize such errors. First, the MRI data in the sixteen continuous slices were acquired with “interleaved” scheme (*i.e.*, the sequence to acquire the data from the 16 contiguous slices is 1 → 3 → 5 ··· 15, 2 → 4 → 6 ··· 16), which introduces an actual interslice gap of 100% to slice thickness. Secondly, spoiling gradients were applied on both sides of the refocusing RF pulses in Slice and Read directions, which would eliminate the unwanted stimulated echoes and minimize the errors in T2 measurement [30, 31]. The reliability of the Bruker MSME protocol for T2 measurement has been validated by both phantom and in-vivo studies [27]. The T2 maps were calculated using Bruker’s Paravision 6.0.1. MRI images and T2 data were analyzed using ImageJ software (https://imagej.nih.gov/ij/). For T2 measurement, oval regions of interest (ROI) of the same size were selected across three consecutive slices in each leg close to the knee bones (Supplementary Fig. 1A). The area of each ROI was 160 pixels (6.1 mm^2^), and they avoided fibula, larger blood vessels, and the subcutaneous fat, and they covered the posterior part of the hindlimb which mainly include gastrocnemius, soleus, and plantaris muscles as shown in the Supplementary Fig. 1B. Because there was no difference in tibia bone volumes between *mdx* and WT mice [32], leg muscle size was measured as the average cross-sectional area (CSA), without subtracting the tibia bone regions in these three consecutive slices.

A single-voxel stimulated echo acquisition-mode (STEAM) MRS sequence was used to measure the fat fraction in the muscles [33, 34]. A voxel of size 2 × 2 × 2 mm^3^ was located in the posterior muscles of the leg for MRS. Localized shimming provided by Bruker was used to minimize the spectral linewidth. The parameters for STEAM were as follows: TR = 2 s, TE = 3 ms, acquisition bandwidth = 10,000 Hz, and acquisition data points = 2048. The routine MRS signal was dominated by the water signal, and an average number of 1 achieved a high signal-to-noise ratio. To enhance the fat signal, the same parameters as the water MRS measurement were used, but variable pulse power and optimized relaxation delay (VAPOR) water suppression was added to the STEAM sequence [35]. To improve the signal-to-noise ratio, the number of averages was increased to 64. The fat fraction was calculated as the fat/water ratio by dividing the magnitude of the fat peak (located at 1000 Hz) by that of the water peak (located at 0 Hz) in the water spectrum (Supplementary Fig. 2) [36].

### EIM measurements

EIM data were obtained from mice placed on a heating pad to maintain consistent body temperature under 1.5% isoflurane anesthesia. The left hindlimb was taped to the measuring surface at an approximately 45° angle extending out from the body. After shaving the hindlimb far and removing the remaining hair with a depilatory cream (Cha.lu.la Disahair cream, GARDEN, Osaka, Japan), calf thickness was measured using a dial thickness gauge (Peacock G-2; Ozaki MFG Co. Ltd., Tokyo, Japan). The skin at the measurement site was pretreated with a skin preparation gel (Nuprep^®^; Weaver and Company, Aurora, CO, USA) to reduce skin impedance. A fixed rigid four-electrode array with a 20 g weight attached to the tip was placed over the gastrocnemius (GC) muscle in the longitudinal direction [37] after applying an electrode cream (SignaCreme^®^; Parker Labs, Fairfield, NJ, USA), which facilitates electrical conduction between the skin and electrodes. A weak electrical current with a wide range of frequencies was applied to the tissues through one set of electrodes, and the resulting voltages were measured via a second set. EIM measurements were performed using an impedance spectroscopy system (mScan-V^™^; Myolex Inc., Boston, MA, USA) to obtain three EIM parameters: reactance (*X*), resistance (*R*), and phase (*arctan X/R*). Multi-frequency measurements over the 1–10,000 kHz range within several seconds were repeated three times and averaged. For the analyses, values from 10 to 1000 kHz were used to avoid both low- and high-frequency artifacts.

### Skeletal muscle histopathology

Mice were anesthetized by intraperitoneal injection of a combination anesthetic (medetomidine 0.3 mg/kg, midazolam 4 mg/kg, and butorphanol 5 mg/kg) and sacrificed by intracardiac perfusion initially with saline, followed by a fixation with 4% paraformaldehyde phosphate buffer solution (163–20145; FUJIFILM Wako Pure Chemical Corp., Osaka, Japan) The hindlimbs including GC muscles were removed and immersed in the same fixative for 24 h at 4 °C. The fixative was then replaced with 0.1 mol/L phosphate buffer solution (pH 7.4) and stored at 4 °C until the dissection of hindlimb GC muscles. The GC muscle tissues were sectioned from the left hindlimb, in which the EIM was measured, and embedded in paraffin for histological analysis. Tissue Sects. (4 μm thick) were stained with hematoxylin and eosin (H&E) for histopathological analysis. The slides were evaluated by a board-certified veterinary pathologist.

To determine the muscle fiber size (CSA) and count, laminin immunohistochemical staining was performed using a BOND RX automated stainer (Leica Biosystems, Deer Park, IL, USA). Transverse sections of muscles (4 μm thick) were stained for the cell membrane with a rat monoclonal anti-laminin 2 alpha antibody (4H8-2, ab11576, dilution 1:1000; Abcam, Cambridge, UK) for 30 min at room temperature. The cell membrane of the muscle fibers was visualized with Polink-2 Plus HRP Rat-NM DAB Detection Kit (D46-6; OriGene Technologies, Inc., Rockville, MD, USA), the Bond Polymer Refine Detection Kit (DS9800; Leica Biosystems, Deer Park, IL, USA), and DAB Enhancer (AR9432; Leica Biosystems, Deer Park, IL, USA). Subsequently, the sections were stained with hematoxylin, dehydrated with 100% ethanol, and mounted using Permount Mounting Medium (SP15-100; Thermo Fisher Scientific, Waltham, MA, USA). Digital images were obtained using a digital slide scanner (NanoZoomer S60; Hamamatsu Photonics, Shizuoka, Japan), and the CSA and muscle fiber count were subsequently analyzed using the image analysis software HALO (Indica Labs, Albuquerque, NM, USA). The analyzed muscle fibers were categorized into three types based on their thickness (small: < 500 μm^2^, middle: 500–1500 μm^2^, large: > 1500 μm^2^) to characterize genotype-related differences.

To evaluate the fibrotic area in the muscles, Sirius red (SR) staining was performed using an automated stainer (DRS2000; Sakura Finetek USA, Inc., Torrance, CA, USA). Dewaxed muscle transverse Sects. (4 μm thick) were stained for collagen with a modified Sirius red staining solution, which consisted of Sirius Red (0.1% w/v, 196–16201; FUJIFILM Wako Pure Chemical Corp., Osaka, Japan), Fast Green FCF (0.1% w/v, 069–00032; FUJIFILM Wako Pure Chemical Corp., Osaka, Japan), and Van Gieson Solution P (224–01405; FUJIFILM Wako Pure Chemical Corp., Osaka, Japan), for 30 min at room temperature. Subsequently, the sections were rinsed with deionized water, dehydrated with 100% ethanol, and mounted using Permount Mounting Medium (SP15-100; Thermo Fisher Scientific, Waltham, MA, USA). Images of the stained sections were captured using a NanoZoomer S60 (Hamamatsu Photonics, Shizuoka, Japan), and areas (μm^2^) of SR-positive and total tissues were analyzed using HALO (Indica Labs, Albuquerque, NM, USA). The SR-positive area (%) was calculated as a measure of the extent of fibrosis excluding the fascia, large tendons, blood vessels, and peripheral nerves from the total tissue area.

### Treatment with a peptide-conjugated PMO

A peptide Pip9b2 (Ac-RXRRBRRFQILYRBRXRB-OH, where ‘X’ was aminohexanoic acid, and ‘B’ was β-alanine)-conjugated PMO (PPMO) [25] that was designed to induce skipping of *Dmd* exon 23 in mice, was manufactured at Takeda Pharmaceutical Company Limited. PPMO was dissolved in saline at a concentration of 2 mg/mL and then warmed for 10 min at 65 °C followed by vortex mixing.

Twenty *mdx* mice at 15 weeks of age were used for pre-EIM recordings. Based on the EIM data, one individual was excluded from the analysis due to exceeding 2 standard deviations (SD) from the mean and then the remaining *mdx* mice were divided into two groups (*n* = 9 for vehicle-treated group, *n* = 10 for PPMO-treated group). Thereafter, the *mdx* mice were intravenously administered PPMO (10 mg/kg) or saline via the tail vein four times every two weeks, starting at 16 weeks of age. To evaluate the drug effect, EIM was recorded twice, at 2 weeks after the first and fourth injections (2W post-dose at 18 weeks of age and 8W post-dose at 24 weeks of age). After completing the 8W post-dose EIM recordings, the animals were sacrificed to obtain GC muscle samples for exon skipping efficiency analysis and dystrophin protein quantification. The samples were stored at -80 °C until use.

### Real-time quantitative polymerase chain reaction (RT-qPCR) and the calculation of exon skipping efficiency

GC muscle tissues were homogenized using a FastPrep-24 5G homogenizer (MP Biochemicals LLC, Irvine, CA, USA) in Lysing Matrix I tubes (6918–100; MP Biochemicals LLC, Irvine, CA, USA) filled with ISOGEN (319–90211; FUJIFILM Wako Pure Chemical Corp., Osaka, Japan). Total RNA was extracted using phenol/chloroform extraction, followed by QuickGene RNA Tissue Kit SII (RT-S2; FUJIFILM Wako Pure Chemical Corp., Osaka, Japan) according to the manufacturer’s instructions. The concentration of total RNA was measured using a microvolume UV–Vis spectrophotometer (NanoDrop 8000; Thermo Fisher Scientific, Waltham, MA, USA) and cDNA was synthesized from 200 ng of total RNA using a High-Capacity cDNA Reverse Transcription Kit (4368813; Invitrogen, Carlsbad, CA, USA) in 20 μL of total reaction volume. RT-qPCR was performed using QuantStudio (Thermo Fisher Scientific, Waltham, MA, USA) and Taqman Gene Expression Master Mix (4369016; Thermo Fisher Scientific, Waltham, MA, USA). One microliter of cDNA solution was used as the template in a 10 μL PCR reaction. The following primers were used for the mouse *Dmd* mRNA containing exons 22, 23, and 24 (non-skipped):

- Forward primer, 5’-CCAAGAAAGCACCTTCAGAAATA-3’;
- Reverse primer 5’-AGGAAAGTTTCTTCCAGTGC-3’; and
- TaqMan probe, 5’- TCTGTCAGAATTTGAAGAGATTGAGGG-3’.

The following primers were used for mouse *Dmd* mRNA containing exons 22 and 24 (exon 23 skipped):

- Forward primer, 5’-CTCTCTGTACCTTATCTTAGTGTTACTG-3’;
- Reverse primer 5’-GGCAGGCCATTCCTCTTT-3’; and
- TaqMan probe, 5’-CTCGGGAAATTACAGAATCACATAAAAACCT-3’.

The following primers were used for mouse glyceraldehyde-3-phosphate dehydrogenase (GAPDH):

- Forward primer, 5’-CCCTGTTCCAGAGACGGCC-3’;
- Reverse primer 5’-GCCAAATCCGTTCACACCGA-3’; and
- TaqMan probe, 5’-CAGTGCCAGCCTCGTCCCGTAGACAAA-3’.

The expression levels of exon 23 non-skipped and exon 23 skipped were normalized to GAPDH expression. The percentage of exon 23 skipping efficiency was calculated using the following formula: expression level of exon 23 skipped / (expression level of exon 23 skipped + expression level of non-skipped) × 100.

### Dystrophin protein analysis

GC muscle tissues were homogenized using a FastPrep-24 5G homogenizer (MP Biochemicals LLC, Irvine, CA, USA) in Lysing Matrix I tubes (6918–100; MP Biochemicals LLC, Irvine, CA, USA) with radioimmunoprecipitation assay (RIPA) buffer (182–02451; FUJIFILM Wako Pure Chemical Corp., Osaka, Japan) containing a protease inhibitor cocktail (P8340; Sigma-Aldrich Corp., Saint Louis, MO, USA) and 5 mM EDTA (347–07481; FUJIFILM Wako Pure Chemical Corp., Osaka, Japan) (modified RIPA buffer). After a 30 min incubation on ice, the homogenates were transferred to new tubes and centrifuged at 12,000 rpm for 20 min at 4 °C. The supernatants were collected and the total protein concentration was adjusted to 4 mg/mL. Dystrophin expression was analyzed with capillary electrophoresis immunoassay using the Wes system (ProteinSimple, San Jose, CA, USA), 66–440 kDa Wes Separation Module (SM-W008; ProteinSimple, San Jose, CA, USA), anti-rabbit detection module (DM-001; ProteinSimple, San Jose, CA, USA), and anti-Dystrophin antibody (ab154168; Abcam, Cambridge, UK) as previously reported [38]. Samples diluted with modified RIPA buffer were loaded into capillaries at a concentration of 0.2 mg/mL of total protein. To calculate the dystrophin expression levels, a standard curve was generated using a mixture of WT mice samples diluted with modified RIPA buffer at a range of concentrations from 0.025 to 0.4 mg/mL of total protein (12.5–200% of dystrophin levels in WT mice). Dystrophin expression values were plotted along a standard curve and calculated as the percentage of dystrophin in the WT mice.

### Data analysis

Statistical analyses were performed using SAS software (SAS Institute Japan, Tokyo, Japan). To compare age-related changes between WT and *mdx* mice, data were assessed using Student’s *t*-test with a closed testing procedure. The comparison was performed at the endpoint at which the maximum difference was predicted. If the resulting two-sided *p*-value was < 0.05, the same comparison was repeated retrospectively to determine the earliest time point with a significant difference. The relationships between 50 kHz EIM reactance and histological parameters (averaged CSA, small fiber frequency, and fibrosis area) were assessed using Pearson’s correlation coefficient. In the drug intervention study, the effect of PPMO in *mdx* mice was evaluated using Student’s *t*-test with a closed testing procedure. Regarding 50 kHz EIM data, if a significant difference was detected between vehicle-treated WT and *mdx* mice, the comparison between vehicle-treated and PPMO-treated *mdx* mice was assessed. To compare the effects between single and repeated treatments, the above analysis was performed using 8W post-dose EIM data, in which the maximum treatment effect was expected. If a significant effect was detected in 8W post-dose EIM, 2W post-dose EIM data were subsequently analyzed. Statistical significance was set at *p* < 0.05. Data are presented as the mean ± standard error of the mean (SEM).

## Results

### Longitudinal changes in muscle structure of *mdx* mice detected by MRI/MRS

**Fig. 1:**
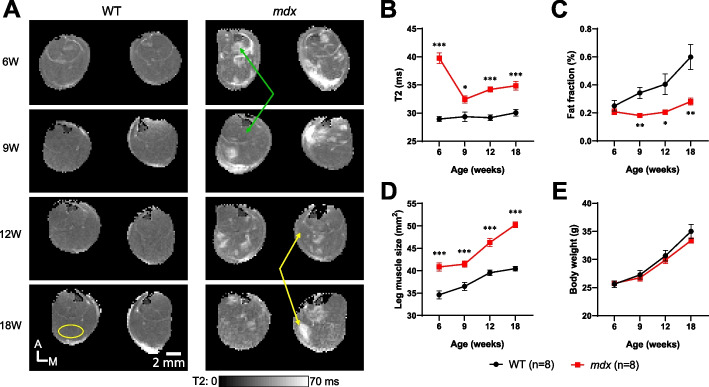
Longitudinal MRI/MRS measurements of muscle T2, leg muscle size, and muscle fat fraction in WT and *mdx* mice. **A** T2 maps from one WT mouse (left) and one *mdx* mouse (right) at 6–18 weeks of age. Areas with high T2 values were consistently observed in both legs of the *mdx* mice at all ages. Green arrows indicate that the region with a high T2 becomes normal at a later time point, whereas yellow arrows indicate that the initially normal T2 region has a high T2 at a later time point. The yellow circle in the bottom-left image shows the ROI definition for the T2 analysis in each slice. A: anterior, M: medial. **B-E** Time course of muscle T2 (**B**), muscle fat fraction (**C**), leg muscle size (**D**), and body weight (**E**) in WT (black circle, *n* = 8) and *mdx* (red square, *n* = 8) mice. Data are represented as mean ± SEM. Statistical comparisons between WT and *mdx* mice were performed retroactively from the endpoint at 18 weeks of age until no significant differences were observed (Student’s *t*-test, ^*^p < 0.05, ^**^p < 0.01, ^***^p < 0.001). MRI: magnetic resonance imaging, MRS: magnetic resonance spectroscopy, T2: the transverse relaxation time constant, WT: wild-type, ROI: regions of interest

To investigate age-related changes in the composition of leg muscles, T2 MRI and MRS data were collected from *mdx* and WT mice at 6–18 weeks of age (Fig. 1). Here, the differences in T2 MRI and MRS data were assumed to indicate changes in the water and fat fractions of the skeletal muscle, respectively. In WT mice, the leg muscle T2 was homogeneous and consistent at different ages (Fig. 1A, left panels). In contrast, regions with elevated T2 were observed in the muscles of both legs in *mdx* mice across all ages (Fig. 1A, right panels). Interestingly, several high T2 regions were observed in different areas of *mdx* mice muscles over time. For instance, the high T2 regions from earlier ages disappeared at later ages (green arrows), and the T2 values of regions that were initially within the normal range increased at later ages (yellow arrows). To quantify the differences in T2, the oval ROIs covering the posterior part of the hindlimb which mainly included gastrocnemius, soleus, and plantaris muscles were set for both legs of each mouse group across the ages. Elevated T2 levels were consistently observed in *mdx* mice at all ages tested, with the highest elevation observed at 6 weeks of age (Fig. 1B, *p* < 0.05). Since elevated T2 could be caused also by a higher fat fraction in the leg muscles, MRS measurements of the fat fraction were performed in these mice. However, the fat fraction in the leg muscles of WT mice, rather than that of *mdx* mice, was higher especially at later ages (Fig. 1C , *p* < 0.05, at 9, 12, and 18 weeks of age). These data suggest that the higher T2 relaxation signal observed in the skeletal muscle of *mdx* mice was not primary due to fat infiltration. Additionally, the T2 changes observed in *mdx* mice were heterogeneous, indicating the degree of edema and inflammation varied across different regions of skeletal muscle and age.

To rule out the possibility of increased muscle mass due to an overgrowth in *mdx* mice, leg muscle size and body weights were compared. Transverse muscle size was assessed using MRI images of the CSA of leg muscle. The overall leg muscle size of *mdx* mice was larger than that of WT mice at all ages tested, with the maximal difference at 18 weeks (Fig. 1D, *p* < 0.001). The body weight of *mdx* mice was not significantly different from that of WT mice (Fig. 1E). These data suggest that the hypertrophic leg muscle in *mdx* mice was due to local muscle pathological changes but not body size change.

### Longitudinal EIM alterations in *mdx* mice

**Fig. 2:**
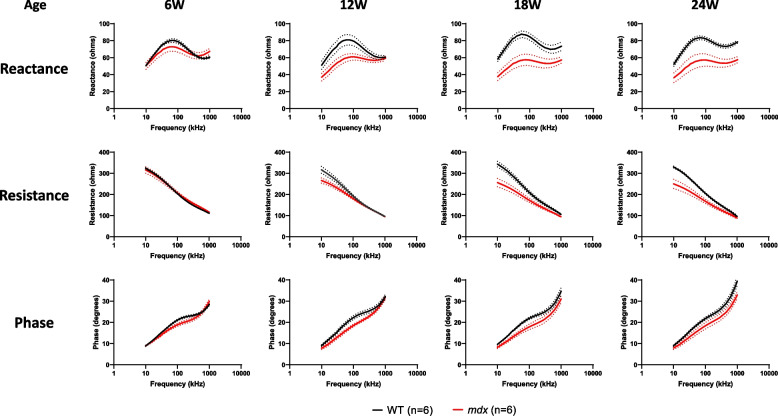
Multifrequency data (10–1000 kHz) for EIM parameters in the natural history study with WT (black line, *n* = 6) and *mdx* (red line, *n* = 6) mice. The EIM data are shown as three major parameters of reactance (top row), resistance (middle row), and phase (bottom row). Data obtained from GC muscle at 6, 12, 18, and 24 weeks of age are shown as mean ± SEM. EIM: electrical impedance myography, WT: wild-type, GC: gastrocnemius, SEM: standard error of the mean

**Fig. 3:**
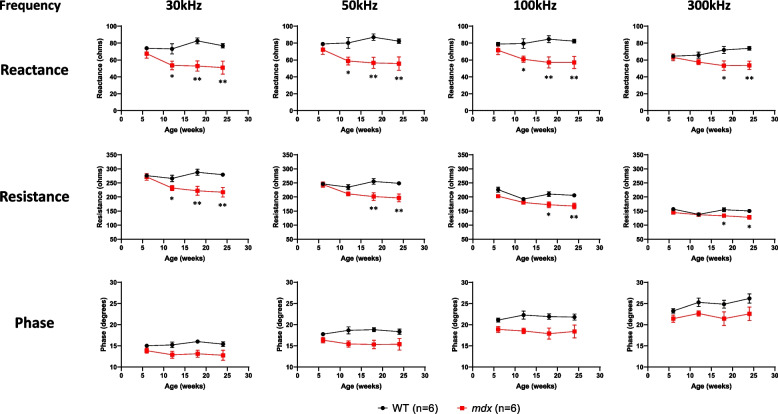
Comparison of EIM data at selected frequencies (30, 50, 100, and 300 kHz) between WT (black circle, *n* = 6) and *mdx* (red square, *n* = 6) mice in a natural history study from 6 to 24 weeks of age. The EIM data are shown as three major parameters of reactance (top row), resistance (middle row), and phase (bottom row). Data at selected frequencies from Fig. 2 are plotted as mean ± SEM. Statistical comparisons between WT and *mdx* mice were performed retroactively from the endpoint at 24 weeks of age until no significant difference was detected (Student’s *t*-test, ^*^p < 0.05, ^**^p < 0.01, ^***^p < 0.001). EIM: electrical impedance myography, WT: wild-type, SEM: standard error of the mean

Because we could replicate the age-dependent changes in muscle composition with MRI [32, 39, 40], we applied longitudinal EIM measurements to WT and *mdx* mice to compare the MRI and EIM data. At 6 weeks of age, there was no significant difference in any EIM parameter between *mdx* and WT mice (Fig. 2, left panels). Among the EIM measures, a clear reduction in reactance was observed in *mdx* mice over a wide range of frequencies at ≥ 12 weeks of age, and this alteration peaked at 18 weeks of age (Fig. 2, top row). A similar trend was observed for resistance, but in the low-frequency range (Fig. 2, middle row). The difference in phase was less evident between *mdx* and WT mice across all ages tested (Fig. 2, bottom row). To quantify the changes in EIM parameters, data at selected frequencies (30, 50, 100, and 300 kHz) were plotted against age (Fig. 3). In WT mice, the longitudinal EIM parameters obtained by repeated measurements showed stable values across the selected frequencies (Fig. 3, black line), suggesting that the method was appropriate for longitudinal EIM evaluation. In contrast, EIM values for reactance and resistance in *mdx* mice exhibited an age-dependent decline (Fig. 3, red line), and the difference between WT and *mdx* mice became significant at 12 weeks of age (*p* < 0.05 for reactance at 30, 50, and 100 kHz, for resistance at 30 kHz) or older (*p* < 0.05 or 0.01 for reactance and resistance at all frequencies evaluated). The EIM value for the phase in *mdx* mice was smaller than that in WT mice at ages ≥ 12 weeks across the selected frequencies, but the difference was not statistically significant. Taken together, these data suggest that EIM parameters can sensitively capture changes in skeletal muscle composition along with dystrophic disease progression.

### Histopathological changes in the GC muscle of *mdx* mice

**Fig. 4:**
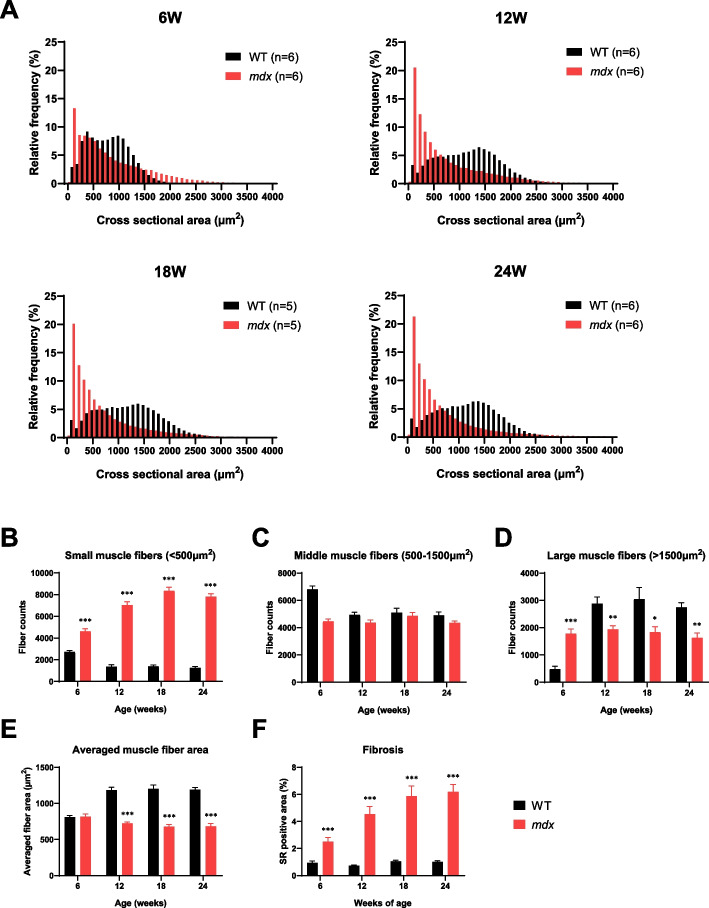
The histopathological analysis of GC muscle at 6–24 weeks of age in WT and *mdx* mice. **A** Comparison of histogram for muscle fiber CSA reveals an obvious difference of muscle composition between WT (black, *n* = 5 or 6) and *mdx* (red, *n* = 5 or 6) mice. Analyzed muscle fiber frequencies from all animals are plotted for each given muscle fiber CSA with a 100 μm^2^-bin. **B-D** The muscle fibers were classified into three types based on thickness (small: < 500 μm^2^, middle: 500–1500 μm^2^, large: > 1500 μm^2^) and compared between genotypes at each time point. **E** The averaged muscle fiber CSA calculated from individual animal values are plotted over time. **F** The fibrosis area quantified as the SR positive area (%) to total muscle area on the GC transverse section are plotted over time. **B-F** Data are represented as mean + SEM (*n* = 5 or 6). Statistical comparisons between WT and *mdx* mice were performed retroactively from the endpoint at 24 weeks of age until no significant difference was detected (Student’s *t*-test, ^*^p < 0.05, ^**^p < 0.01, ^***^p < 0.001). GC: gastrocnemius, WT: wild-type, CSA: cross-sectional area, SR: Sirius red, SEM: standard error of the mean

To gain further insights into the difference in muscle fiber size between WT and *mdx* mice, morphological analysis by laminin staining of GC muscle fibers across ages was performed using a different set of animals from the longitudinal EIM study. In WT mice at 6 weeks of age, the histogram of CSAs of individual muscle fibers showed a bimodal-like pattern with peaks at approximately 500 and 1000 μm^2^ (Fig. 4A, black, top left). At 12 weeks of age, the histogram of CSAs changed into a wider distribution pattern, with a peak at approximately 1400 μm^2^ (Fig. 4A, black, top right), and this pattern persisted at 18 and 24 weeks of age (Fig. 4A, black, bottom). In contrast, the histogram of CSA in *mdx* mice at 6 weeks of age showed a skewed-right pattern with a peak at approximately 100 μm^2^ (Fig. 4A, red, top left). The skewed-right distribution of the CSA pattern was consistently observed at 12, 18, and 24 weeks of age, with a further increase in the relative frequency of CSA under 500 μm^2^ (Fig. 4A, red). To quantify the characteristics of CSA distribution, the size of muscle fibers was classified into three types based on the thickness (small: < 500 μm^2^, middle: 500–1500 μm^2^, large: > 1500 μm^2^) (Fig. 4B-D). The number of small muscle fibers was significantly higher in *mdx* mice than in WT mice in an age-dependent manner (Fig. 4B, *p* < 0.001 at all ages), whereas there was no significant difference in the number of middle muscle fibers (Fig. 4C). The number of large muscle fibers was significantly higher in *mdx* mice than in WT mice at 6 weeks of age (*p* < 0.001), but this relationship was reversed at ages ≥ 12 weeks (*p* < 0.05 or 0.01, Fig. 4D). These differences in muscle fiber size and distribution resulted in a significantly smaller average of individual muscle fiber CSA within the GC muscle in *mdx* mice compared to WT mice at ≥ 12 weeks of age (Fig. 4E, *p* < 0.001). These data suggest that the age-dependent changes in GC muscle fiber morphology in *mdx* mice are more closely associated with changes in EIM parameters rather than T2 MRI.

In addition to muscle fiber morphology, collagenous connective tissue was stained with SR to quantify muscle fibrosis in both WT and *mdx* mice. The fibrotic area in the GC muscle did not change until 24 weeks of age in WT mice, while that in *mdx* mice was already significantly increased at 6 weeks of age compared to WT mice, followed by a further increase with age (Fig. 4F, *p* < 0.001 at all ages). Taken together, these data suggest that *mdx* mice show a parallel progression of increased small muscle fiber and fibrosis area in the GC muscle.

### Correlation between EIM reactance and histological data in GC muscle

**Fig. 5:**
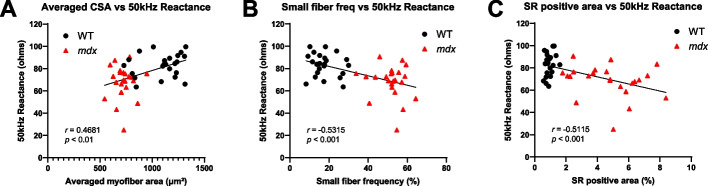
Relationship between EIM reactance and histopathological parameters in the GC muscle at 6–24 weeks of age in WT (black circle, *n* = 23) and *mdx* (red triangle, *n* = 23) mice. EIM data were obtained before muscle sampling at each time point. Correlations between 50 kHz EIM reactance and averaged muscle fiber CSA (**A**), the small muscle fiber population (CSA < 500 μm^2^) (**B**), or SR positive fibrosis area (**C**) were analyzed. Data were assessed using the Pearson’s correlation coefficient. EIM: electrical impedance myography, GC: gastrocnemius, WT: wild-type, CSA: cross-sectional area, SR: Sirius red

The EIM parameters in the set of animals used for morphological assays were comparable to those obtained in the longitudinal EIM study (Supplementary Fig. 3). We then analyzed the relationship between the most sensitive EIM parameter of 50 kHz reactance and histological muscle data (Fig. 5). The average muscle fiber area in the GC muscle exhibited a significant positive correlation with EIM reactance (Fig. 5A; *r* = 0.4681, *p* < 0.01). In contrast, both the frequency of small muscle fibers (Fig. 5B; *r* = -0.5315, *p* < 0.001) and the fibrosis area of the GC muscle (Fig. 5C; *r* = -0.5115, *p* < 0.001) showed significant negative correlations with EIM reactance. These data suggest that changes in EIM reactance are associated with both muscle fiber atrophy and increased fibrosis in skeletal muscle.

### Effects of exon skipping by PPMO treatment on EIM reactance in *mdx* mice

**Fig. 6:**
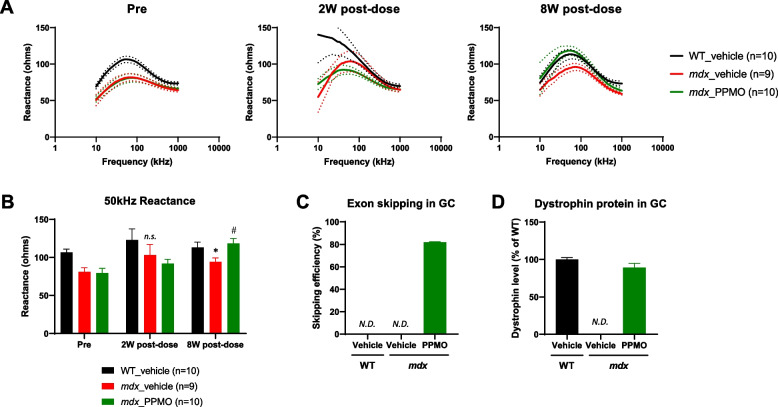
Effects of single and repeated intravenous administration with an exon skipper PPMO (10 mg/kg, 4 times every 2 weeks) on EIM reactance in *mdx* mice. **A** The multifrequency data (10–1000 kHz) for EIM reactance in vehicle-treated WT mice (black line, *n* = 10), vehicle-treated (red line, *n* = 9), or PPMO-treated *mdx* mice (green line, *n* = 10) during the pre, 2W post-dose (2 weeks after the 1st administration), and 8W post-dose (2 weeks after the 4th administration) measurements. **B** Summarized 50 kHz EIM reactance before and after single and repeated administration of PPMO (Pre, 2W post-dose, and 8W post-dose). Statistical comparisons were performed using Student’s *t*-test with a closed testing procedure (^*^p < 0.05, *n.s.*: not significant vs. the vehicle-treated WT mice group, ^#^*p* < 0.05 vs. the vehicle-treated *mdx* mice group). **C** RT-qPCR for calculation of exon skipping efficiency in the GC muscle. **D** Capillary electrophoresis immunoassay using the Wes system for quantification of dystrophin protein levels in the GC muscle. The percentage of exon skipping efficiency was calculated using the following formula: expression level of exon 23 skipped / (expression level of exon 23 skipped + expression level of non-skipped) × 100. Dystrophin expression value was calculated as a percentage of WT mice dystrophin levels. *N.D.*: not detected. Data are represented as mean ± SEM. PPMO: peptide (Pip9b2)-conjugated antisense phosphorodiamidate morpholino oligomer, EIM: electrical impedance myography, WT: wild-type, RT-qPCR: real-time quantitative polymerase chain reaction, GC: gastrocnemius, SEM: standard error of the mean

The EIM data suggested that the sensitivity of EIM reactance was adequate to detect changes in muscle composition in *mdx* mice. To investigate whether EIM could detect changes in muscles following dystrophin restoration in *mdx* mice, a drug intervention study with an exon skipper PPMO was conducted. Prior to PPMO administration at 16 weeks of age, *mdx* mice (*n* = 20) showed a decrease in all EIM parameters compared to WT mice (*n* = 10), as observed in the natural history study (Supplementary Fig. 6A). At 50 kHz frequency, EIM reactance in *mdx* mice was significantly lower than that in WT mice (Supplementary Fig. 6B, *p* < 0.001). With the exception of one outlier, *mdx* mice were equally allocated to the vehicle-treatment (*n* = 9) and PPMO-treatment groups (*n* = 10) based on pre-EIM values (Fig. 6A, B, Pre). After then, mice were intravenously administered vehicle or PPMO at 10 mg/kg every two weeks for 8 weeks (4 doses total). At 18 weeks of age, 2 weeks after the first administration, *mdx* mice still showed a decrease in reactance compared to WT mice in the vehicle-treated group (Fig. 6A, 2W post-dose, black and red lines). However, the quantitated 50 kHz reactance was not significantly different between *mdx* mice and WT mice treated with vehicle because of the large variance (Fig. 6B, 2W post-dose). At 24 weeks of age, 8 weeks after starting repeated administration, *mdx* mice still showed a decrease in reactance across frequencies compared to WT mice in the vehicle-treated groups (Fig. 6A, 8W post-dose, black and red lines). In contrast, the reactance in *mdx* mice treated with PPMO was comparable to that in vehicle-treated WT mice (Fig. 6A, 8W post-dose, black and green lines). The quantitated 50 kHz reactance data showed that the reactance in *mdx* mice was significantly lower than that in WT mice in the vehicle-treated groups (*p* < 0.05), whereas the reactance in *mdx* mice treated with PPMO was significantly higher than that in *mdx* mice treated with vehicle (*p* < 0.05, Fig. 6B, 8W post-dose). The pharmacological effects of PPMO were further substantiated by the assessment of exon skipping efficiency and dystrophin protein expression in WT and *mdx* mice, in which EIM was measured. Repeated treatment with PPMO induced 81.9 ± 0.5% of exon 23 skipping efficiency in *mdx* mice compared with WT mice, while the exon skipping efficiency could not be evaluated in WT and *mdx* mice treated with vehicle due to the detection limit (Fig. 6C). As a consequence of the increased exon skipping, muscular dystrophin protein in *mdx* mice treated with PPMO was restored to 89.2 ± 5.6% of that in WT mice (Fig. 6D). These data indicate that EIM reactance could detect the restoration of abnormalities in dystrophic muscle due to an increase in functional dystrophin protein after treatment with exon skippers.

## Discussion

To advance the practical application of EIM as a translational biomarker for DMD research, we investigated the sensitivity of EIM to disease progression and drug responses in *mdx* mice. Our findings provide evidence that mouse EIM reactance can detect structural and compositional alterations in skeletal muscle tissues resulting from dystrophin deficiency or its restoration through pharmacological interventions.

The two *mdx* mouse strains are well-known: the C57BL/10 strain *mdx* (B10-*mdx*) mouse and the D2.B10-*Dmd*^*mdx*^/J (D2-*mdx*) mouse. B10-*mdx* mouse is widely used as an animal model for DMD research [6, 7, 41, 42]. D2-*mdx* mice, created by backcrossing mice onto a DBA/2 J background, have been reported to exhibit decreases in EIM, similar to B10-*mdx* mice [43, 44], but this strain displayed a more severe disease phenotype, including decreased muscle mass, muscle weakness, severe fibrosis, and fat accumulation compared to B10-*mdx*. These phenotypes in D2-*mdx* mice may recapitulate several human characteristics of DMD myopathy [45–47]. However, the pronounced calcifications observed in skeletal muscles from an early age (7–10 weeks) in D2-*mdx* [46, 47] are distinctly different from the phenotypes observed in human DMD myopathy. Since calcification in skeletal muscles was predicted to directly affect EIM parameters, B10-*mdx* mice without calcification in skeletal muscles were selected for this study to investigate the contribution of dystrophin-related muscular changes to EIM alterations.

MRI T2 imaging is a valuable tool that enables the assessment of muscle composition, including damage, inflammation, and fat infiltration, in dystrophic muscle [18]. The highest T2 elevation was observed at 6 weeks of age in *mdx* mice (Fig. 1B), which is consistent with the findings of previous reports [32, 39, 40]. In addition, MRI T2 images demonstrated that *mdx* mice had muscle heterogeneity consisting of separated muscle regions with different T2 dynamic processes (Fig. 1A, green/yellow arrows). These results indicate that repeated pathological changes, such as inflammation and edema, resulted in the emergence of ectopic high T2 muscle regions from 6 to 18 weeks of age in *mdx* mice. In clinical studies, muscle T2 in patients with DMD is reportedly increased by fat infiltration, in addition to muscle damage, inflammation, and edema [34, 48, 49]. However, in the present study, ^1^H-MRS measurements revealed a lower fat fraction in the hindlimb muscle of *mdx* mice (Fig. 1C), consistent with a previous report [50]. This finding suggests that the replacement of muscles by fat tissue, as observed in patients with DMD and associated with post-inflammatory muscular atrophy, does not occur in *mdx* mice, at least at these ages. Given that *mdx* mice show an increased inflammatory response when young [6–9], the higher T2 observed in the skeletal muscle of *mdx* mice mainly reflects edema associated with inflammation [32, 33]. This conclusion is also supported by our histological finding with H&E staining in which myofiber degeneration/regeneration and mononuclear/inflammatory cell infiltration were observed in the hindlimb muscle of *mdx* mice at 6 weeks of age (Supplementary Fig. 4B).

In the comparison of longitudinal data between EIM and MRI, there was no significant difference in EIM parameters between WT and *mdx* mice at 6 weeks of age, when the maximum T2 elevation was observed in the MRI study (Figs. 2 and 3). Subsequently, a significant reduction in EIM reactance was observed in *mdx* mice, peaking at 18 weeks of age. The reduction in EIM in *mdx* mice observed in this study is consistent with the results of a previous study [51]. The analysis of individual muscle fibers with laminin staining provided supportive data for understanding the difference in the sensitivity of T2 MRI signals and EIM reactance to disease progression in *mdx* mice. Specifically, age-dependent alterations in muscle fibers in *mdx* mice were significantly different from those in WT mice (Fig. 4). The time course of the decline in averaged muscle fiber area detected at 12 weeks of age was similar to that of EIM reactance (Figs. 3 and 4E). The morphological changes in muscle fibers appeared to follow the inflammatory response with some delay because the averaged myofiber size had not yet changed in *mdx* mice at 6 weeks of age (Fig. 4E), which exhibited a significant increase in T2 (Fig. 1B). The direct correlation between EIM reactance and muscle fiber size (Figs. 5A, B), in contrast to the T2 MRI signal that peaks at 6 weeks and then decline, suggests that EIM reactance is useful to detect morphological changes in muscle fibers following an inflammatory response during disease progression in *mdx* mice. Furthermore, the increased fibrotic area was associated with a reduction in EIM reactance (Fig. 5C), which corresponds to a previous report [51]. Skeletal muscle fibrosis in DMD, in which muscle fibers are damaged by inflammation and eventually replaced by connective tissues, contributes to progressive muscle weakness through the loss of muscular contractility and flexibility [52]. In the present study, although a moderate increase in the fibrotic area was already observed in *mdx* mice at 6 weeks of age, the difference between WT and *mdx* mice became more significant at ≥ 12 weeks of age (Fig. 4F) when a reduction in EIM reactance was observed in *mdx* mice. In addition, the increased fibrosis area was well associated with the increased number of small muscle fibers in *mdx* mice (Fig. 4B, F). These findings suggest that EIM reactance is also sensitive to connective tissue deposition associated with downsizing myofibers in *mdx* mice.

EIM resistance also decreased in an age-dependent manner, and the EIM phase showed a declining trend in *mdx* mice throughout the measurement period (Figs. 2 and 3). The larger leg muscle size (Fig. 1D and Supplementary Fig. 5B), lower fat fraction (Fig. 1C), and fibrosis progression (Fig. 4F) observed in *mdx* mice are potential causal factors for the decline in EIM resistance from 12 weeks of age. Because hypertrophic muscle, decreased fat, and accumulation of connective tissue enhance electrical conductivity in muscle, which results in a decrease in EIM resistance. In clinical studies, EIM detected a progressive increase in resistance [17] or significant decreases in phase [13–15] in boys with DMD compared to healthy controls. This discrepancy from our results may be attributed to the fact that *mdx* mice do not show a reduction in muscle volume or fat substitution with disease progression, unlike patients with DMD. Patients with DMD show a significant increase in EIM resistance associated with muscle volume loss and intramuscular fat deposition [17]. As for the EIM phase, as this angle is determined by both resistance and reactance (phase angle = *arctan* reactance / resistance), the change in phase may have attenuated by the both reductions in resistance and reactance in *mdx* mice. Therefore, in translational research with EIM, it is important to consider the differences between *mdx* mice and patients with DMD and use each parameter properly.

In the pharmacological intervention study, we could not assess the effect of PPMO on EIM 2 weeks after a single dose because the EIM decline disappeared in *mdx* mice (Fig. 6A, B, 2W post-dose). This might be attributable to the short interval from the pre-measurement period (3 weeks). Although EIM is a noninvasive measurement method, skin pretreatment to improve measurement accuracy may cause slight scratches on the vulnerable skin surface in animals. Therefore, it may be necessary to ensure a sufficient measurement interval to allow recovery from skin damage after this procedure. Indeed, a 6-week interval between 2 and 8W post-dose EIM measurements appeared to be sufficient for recovery from skin damage, resulting in the successful detection of the efficacy of PPMO, along with slight variations in EIM data (Fig. 6 A, B, 8W post-dose). Consecutive EIM recordings from the same animal may be necessary to establish sufficient intervals for precise measurements.

Repeated administration of PPMO increased the EIM reactance in *mdx* mice (Fig. 6 A, B, 8W post-dose). Notably, the reduced EIM reactance in *mdx* was reversed to the level of that in WT with PPMO intervention. After repeated administration of PPMO, the exon 23 skipping efficacy and the ensuing functional dystrophin protein production in *mdx* muscle were maintained at a higher level of 80–90% (Fig. 6C, D). In this study, we did not confirm by histopathology whether increased dystrophin could improve diseased myofibers and connective tissue in *mdx* muscle because we prioritized the measurement of dystrophin protein/exon skipping efficacy by using these muscle tissues. Functional dystrophin produced by exon skipping reduced centrally nucleated muscle fibers in *mdx* mice [23], suggesting that the increased dystrophin observed in this study could alleviate the accelerated degeneration-regeneration cycle in dystrophic myofibers [53]. Furthermore, a significant expression of micro-dystrophin in the skeletal muscles in *mdx* mice with the adeno-associated virus systemic gene transfer therapy resulted in not only a reduction in centrally nucleated fibers but also a normalization of muscle fiber size [54]. These findings, coupled with the close association between EIM alterations and changes in muscle composition, support the PPMO dosing regimen used in this study could normalize skeletal muscle abnormalities in *mdx* mice. Further studies regarding the dose-dependency and intervention period of PPMO are required to investigate the amount and time course of dystrophin protein that would be adequate to recover from muscle composition abnormalities in *mdx* mice.

## Conclusions

Our findings suggest that muscle T2 MRI signals have the potential to detect early inflammatory responses associated with dystrophin deficiency, whereas EIM parameters can respond to subsequent myofiber size reduction and/or connective tissue deposition in the skeletal muscle during disease progression. Furthermore, the pharmacological intervention study using PPMO demonstrated that EIM was responsive to changes in the skeletal muscle composition with deficiency or restoration of dystrophin. To the best of our knowledge, this is the first study to confirm EIM response in *mdx* mice by antisense-mediated exon skipping. Given the established utility of EIM in monitoring disease progression in patients with DMD, taken together with our results, we conclude that EIM reactance has the potential to serve as a valuable and translatable biomarker for monitoring the restoration of dystrophin-deficient muscle abnormalities by exon skippers in clinical studies of DMD.

### Supplementary Information

**Additional file 1: Supplementary Figure 1. **Definition of the oval ROIs in the three axial slices for quantitative T2 analysis. **A** Based on the first TE image from the MSME MRI scan, the three slices immediately below the knee bones were selected for ROI placements as shown by the yellow ovals. **B** T2 maps with the yellow oval ROIs show that these ROIs avoid fibula, larger blood vessels, and the subcutaneous fat. Each ROI is 160 pixels (6.1 mm^2^) and covers the posterior part of the hindlimb which mainly include gastrocnemius, soleus, and plantaris muscles. ROI: regions of interest, T2: transverse relaxation time constant, TE: echo time, MSME: multi-slice multi-echo, MRI: magnetic resonance imaging.

**Additional file 2: Supplementary Figure 2. **MRS measurement of fat fraction in leg muscles. **A** Non-water-suppressed spectrum from the 2 × 2 × 2 mm^3^ voxel, as shown in the leg muscle in the inset figure. The typical peak from tissue water was set to 0 Hz. No clear peak was observed at the fat position (1000 Hz). **B** Water-suppressed spectrum from the same voxel demonstrates a fat peak around the typical position of 1000 Hz. Note the different scaling of the y axis. The fat fraction was calculated by dividing the amplitude of the fat peak by the water amplitude (Fig. A). MRS: magnetic resonance spectroscopy.

**Additional file 3: Supplementary Figure 3. **The multifrequency data (10–1000 kHz) of EIM parameters in WT (black line) and *mdx* (red line) mice from different groups and ages of animals. The EIM data are presented as three major parameters of reactance (top row), resistance (middle row), and phase (bottom row). Data were obtained from 5 or 6 animals in each group before tissue sampling for histopathological analysis (Fig. 4) at 6, 12, 18, or 24 weeks of age. Data are represented as mean ± SEM. EIM: electrical impedance myography, WT: wild-type, SEM: standard error of the mean.

**Additional file 4: Supplementary Figure 4. **Histopathology of the GC muscle at 6 weeks of age in WT and *mdx* mice. H&E analysis revealed typical alterations as dystrophic histopathology. The GC muscle from WT mice exhibited typical morphology (**A**). In *mdx* mice, myofiber degeneration/necrosis (minimal to mild), inflammatory cell infiltration (mild and rare) (indicated by arrows in **B**), myofiber regeneration (**C**; minimal to mild), myofiber central nuclei (**D**; moderate to marked), mononuclear cell infiltration (**E**; minimal to mild), and myofiber mineralization (indicated by arrows in **F**; minimal) were observed at 6 weeks of age and continued to be present until 24 weeks of age.

**Additional file 5: Supplementary Figure 5. **Comparison of body weight (**A**) and hindlimb muscle thickness (**B**) between WT and *mdx* mice in the EIM natural history study. Data were obtained from WT (black,* n *= 6) and *mdx* (red,* n *= 6) mice using consecutive EIM measurements (Figs. 2, 3) at 6–24 weeks of age. Data are represented as mean + SEM. Statistical comparisons between WT and *mdx* mice were performed retroactively from the endpoint at 24 weeks of age until no significant differences were observed (Student’s *t*-test, ^*^p < 0.05, ^**^p < 0.01, ^***^p < 0.001). WT: wild-type, EIM: electrical impedance myography, SEM: standard error of the mean.

**Additional file 6: Supplementary Figure 6. **Comparison of all EIM parameters between WT and *mdx* mice before administration with PPMO. **A** The multifrequency data (10-1000 kHz) for the EIM reactance, resistance, and phase in WT (black line, *n* = 10) and *mdx* (red line, *n* = 20) mice in the pre EIM measurements before grouping. **B** Comparison of 50 kHz EIM parameters between WT (black, *n* = 10) and *mdx* (red, *n* = 20) mice before grouping. Statistical comparisons were performed by Student’s *t*-test (reactance: ^***^p < 0.001, resistance: ^***^p < 0.001, phase: ^**^p < 0.01). Data are represented as mean ± SEM. EIM: electrical impedance myography, WT: wild-type, PPMO: peptide (Pip9b2)-conjugated antisense phosphorodiamidate morpholino oligomer, SEM: standard error of the mean.

**Additional file 7: Supplementary Tables. **Summary of statistical analyses in this study. Statistical analyses were performed using SAS software. To compare parameters between WT and *mdx* mice or vehicle-treatment and PPMO treatment, data were assessed using Student’s *t*-test with a closed testing procedure. The *p*-values observed form the analyses shows in the tables. WT: wild type, PPMO: peptide (Pip9b2)-conjugated antisense phosphorodiamidate morpholino oligomer.

## Data Availability

All data generated or analyzed during this study are included in the published article [and its supplementary information files].

## Abbreviations

ASO: Antisense oligonucleotide
CSA: Cross-sectional area
DMD: Duchenne muscular dystrophy
EIM: Electrical impedance myography
FLASH: Fast low-angle shot
GC: Gastrocnemius
H&E: Hematoxylin and eosin
IACUC: Institutional Animal Care and Use Committee
mdx: Muscular dystrophy X-linked mouse
MRI: Magnetic resonance imaging
MRS: Magnetic resonance spectroscopy
MSME: Multi-slice multi-echo
PPMO: Peptide (Pip9b2)-conjugated antisense phosphorodiamidate morpholino oligomer
ROI: Regions of interest
RT-qPCR: Real-time quantitative polymerase chain reaction
SEM: Standard error of the mean
SR: Sirius red
STEAM: Single-voxel stimulated echo acquisition mode
T2: Transverse relaxation time constant
TE: Echo time
TR: Repetition time
VAPOR: Variable pulse power and optimized relaxation delays
WT: Wild-type
