# Supplementary figures and images for: Electrical impedance myography detects dystrophin-related muscle changes in mdx mice
Source: Skelet Muscle. 2023 Nov 18;13:19. doi: 10.1186/s13395-023-00331-1 (PMC10657153; doi:10.1186/s13395-023-00331-1)

## Slide 1
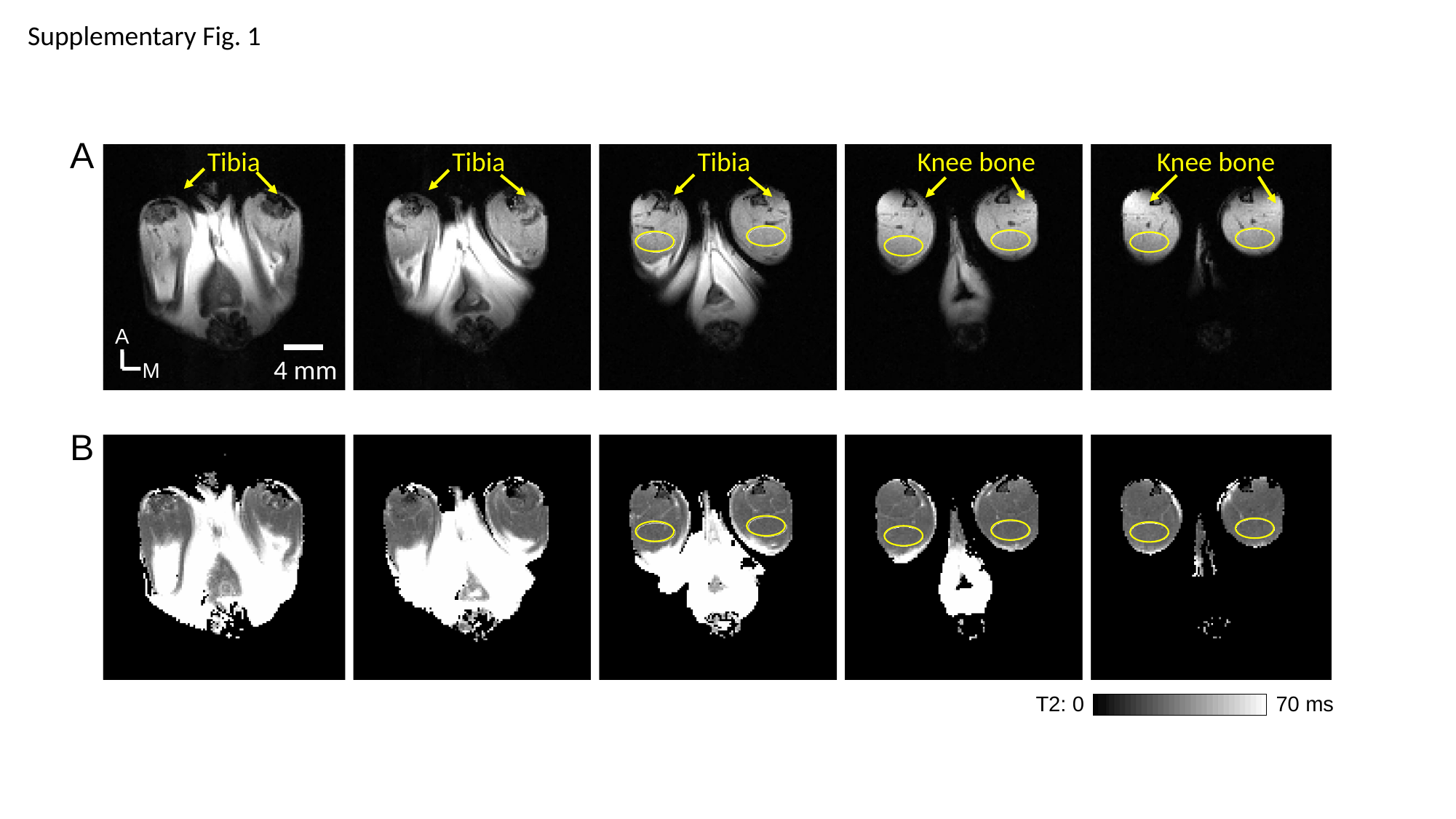

Supplementary Fig. 1
A
Knee bone
Tibia
Tibia
Knee bone
Tibia
A
4 mm
M
B
70 ms
T2: 0

Supplement: Supplementary file 1 — Additional file 1: Supplementary Figure 1. Definition of the oval ROIs in the three axial slices for quantitative T2 analysis. A Based on the first TE image from the MSME MRI scan, the three slices immediately below the knee bones were selected for ROI placements as shown by the yellow ovals. B T2 maps with the yellow oval ROIs show that these ROIs avoid fibula, larger blood vessels, and the subcutaneous fat. Each ROI is 160 pixels (6.1 mm2) and covers the posterior part of the hindlimb which mainly include gastrocnemius, soleus, and plantaris muscles. ROI: regions of interest, T2: transverse relaxation time constant, TE: echo time, MSME: multi-slice multi-echo, MRI: magnetic resonance imaging. [file 13395_2023_331_MOESM1_ESM.pptx]

## Slide 1
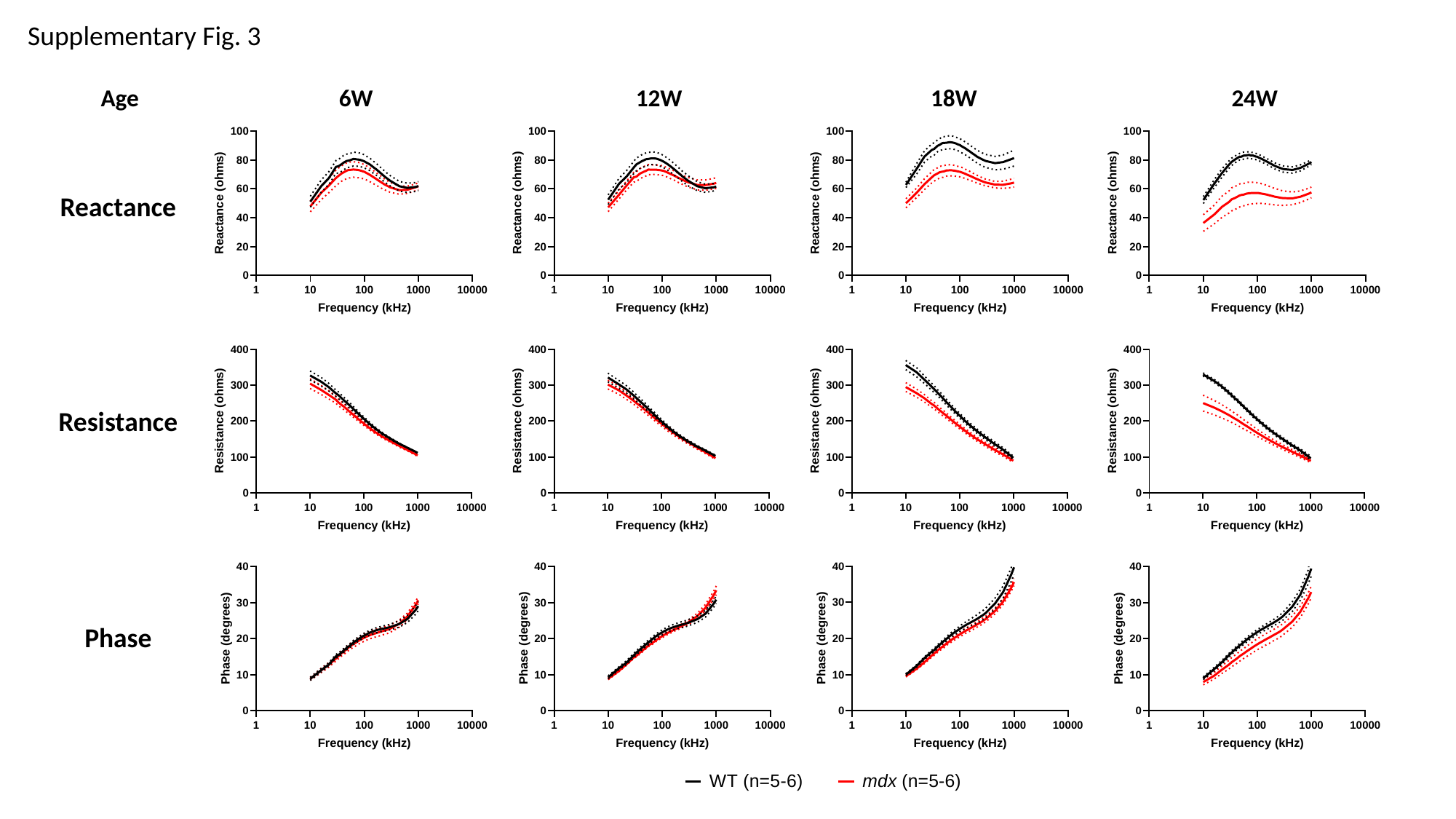

Supplementary Fig. 3
Age
6W
12W
18W
24W
Reactance
Resistance
Phase

Supplement: Supplementary file 3 — Additional file 3: Supplementary Figure 3. The multifrequency data (10–1000 kHz) of EIM parameters in WT (black line) and mdx (red line) mice from different groups and ages of animals. The EIM data are presented as three major parameters of reactance (top row), resistance (middle row), and phase (bottom row). Data were obtained from 5 or 6 animals in each group before tissue sampling for histopathological analysis (Fig. 4) at 6, 12, 18, or 24 weeks of age. Data are represented as mean ± SEM. EIM: electrical impedance myography, WT: wild-type, SEM: standard error of the mean. [file 13395_2023_331_MOESM3_ESM.pptx]

## Slide 1
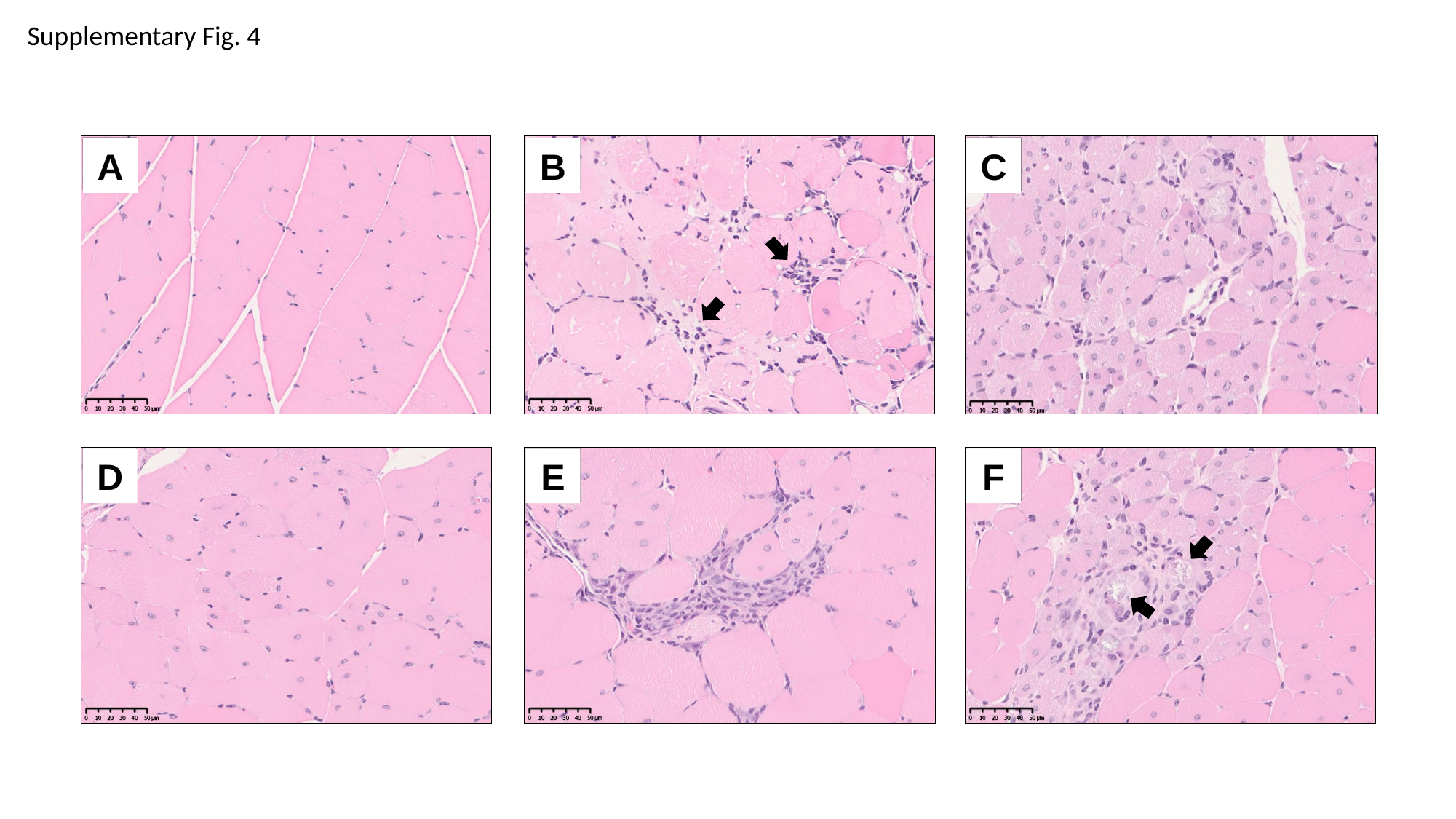

Supplementary Fig. 4
A
B
C
D
E
F

Supplement: Supplementary file 4 — Additional file 4: Supplementary Figure 4. Histopathology of the GC muscle at 6 weeks of age in WT and mdx mice. H&E analysis revealed typical alterations as dystrophic histopathology. The GC muscle from WT mice exhibited typical morphology (A). In mdx mice, myofiber degeneration/necrosis (minimal to mild), inflammatory cell infiltration (mild and rare) (indicated by arrows in B), myofiber regeneration (C; minimal to mild), myofiber central nuclei (D; moderate to marked), mononuclear cell infiltration (E; minimal to mild), and myofiber mineralization (indicated by arrows in F; minimal) were observed at 6 weeks of age and continued to be present until 24 weeks of age. [file 13395_2023_331_MOESM4_ESM.pptx]

## Slide 1
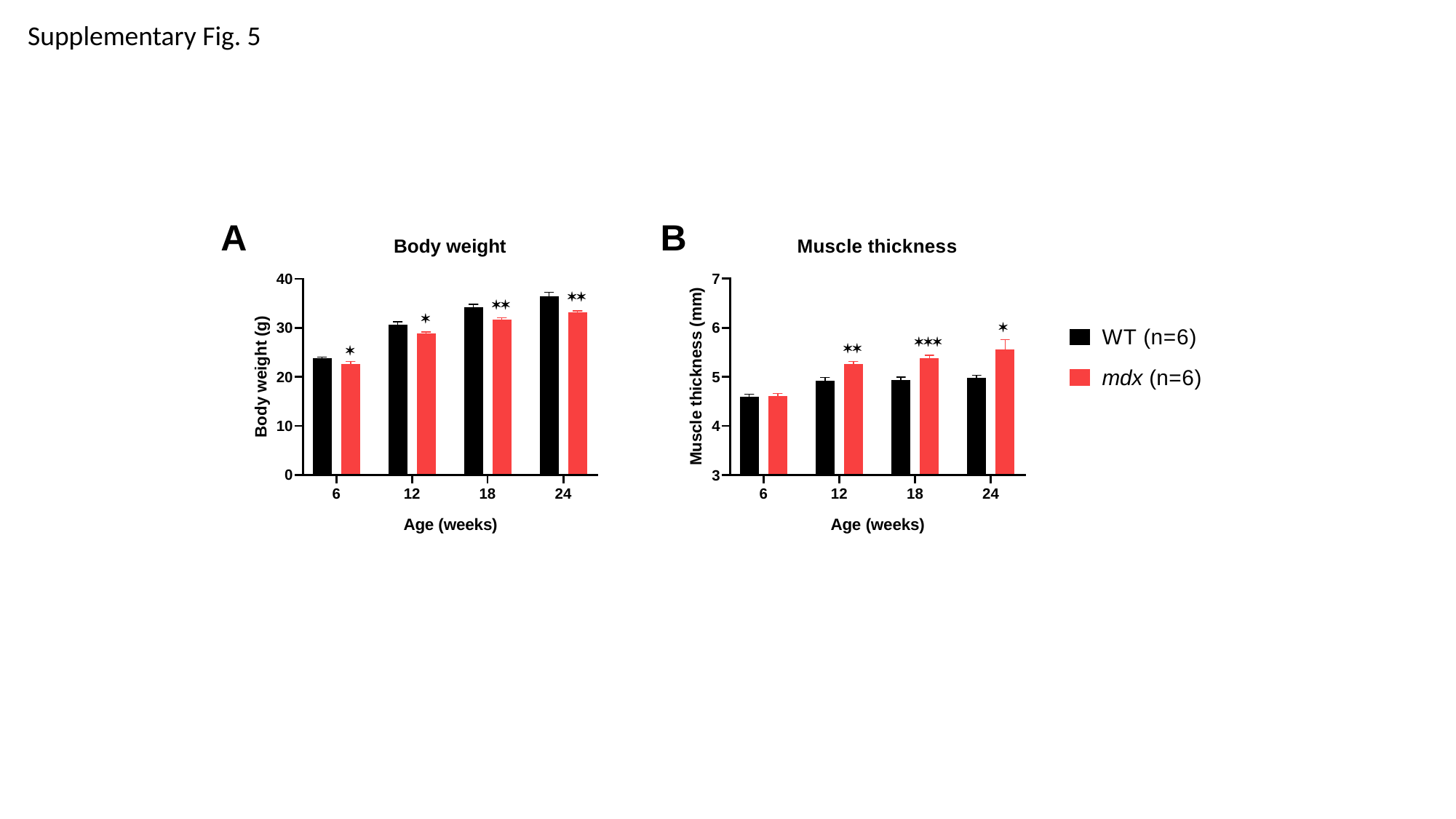

Supplementary Fig. 5
A
B
**
**
*
*
***
**
*

Supplement: Supplementary file 5 — Additional file 5: Supplementary Figure 5. Comparison of body weight (A) and hindlimb muscle thickness (B) between WT and mdx mice in the EIM natural history study. Data were obtained from WT (black, n = 6) and mdx (red, n = 6) mice using consecutive EIM measurements (Figs. 2, 3) at 6–24 weeks of age. Data are represented as mean + SEM. Statistical comparisons between WT and mdx mice were performed retroactively from the endpoint at 24 weeks of age until no significant differences were observed (Student’s t-test, *p < 0.05, **p < 0.01, ***p < 0.001). WT: wild-type, EIM: electrical impedance myography, SEM: standard error of the mean. [file 13395_2023_331_MOESM5_ESM.pptx]

## Slide 1
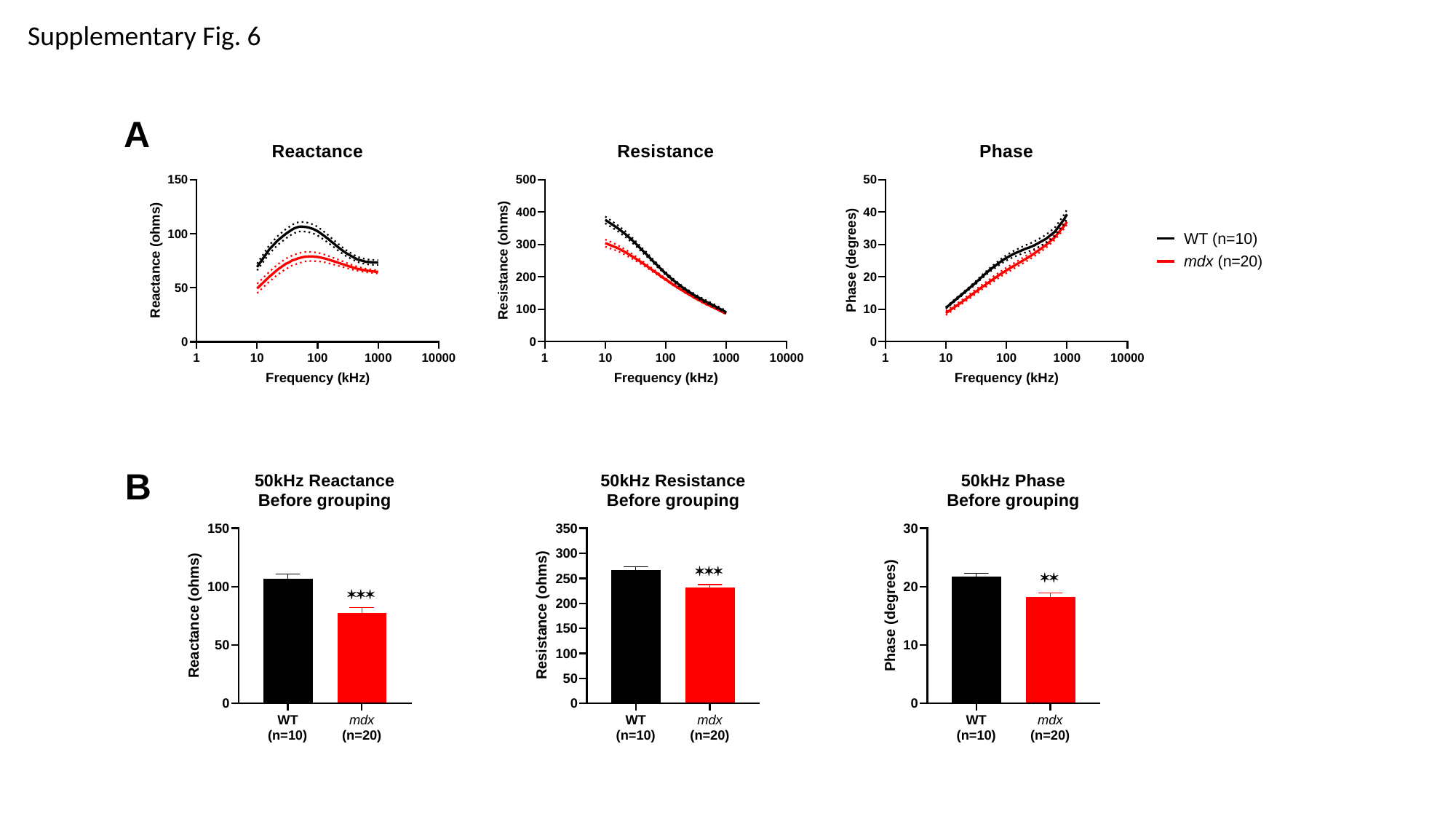

Supplementary Fig. 6
A
B
***
**
***

Supplement: Supplementary file 6 — Additional file 6: Supplementary Figure 6. Comparison of all EIM parameters between WT and mdx mice before administration with PPMO. A The multifrequency data (10-1000 kHz) for the EIM reactance, resistance, and phase in WT (black line, n = 10) and mdx (red line, n = 20) mice in the pre EIM measurements before grouping. B Comparison of 50 kHz EIM parameters between WT (black, n = 10) and mdx (red, n = 20) mice before grouping. Statistical comparisons were performed by Student’s t-test (reactance: ***p < 0.001, resistance: ***p < 0.001, phase: **p < 0.01). Data are represented as mean ± SEM. EIM: electrical impedance myography, WT: wild-type, PPMO: peptide (Pip9b2)-conjugated antisense phosphorodiamidate morpholino oligomer, SEM: standard error of the mean. [file 13395_2023_331_MOESM6_ESM.pptx]
