# Supplementary material for: Electrical impedance myography detects dystrophin-related muscle changes in mdx mice
Source: Skelet Muscle. 2023 Nov 18;13:19. doi: 10.1186/s13395-023-00331-1 (PMC10657153; doi:10.1186/s13395-023-00331-1)
Supplement: Supplementary file 2 — Additional file 2: Supplementary Figure 2. MRS measurement of fat fraction in leg muscles. A Non-water-suppressed spectrum from the 2 × 2 × 2 mm3 voxel, as shown in the leg muscle in the inset figure. The typical peak from tissue water was set to 0 Hz. No clear peak was observed at the fat position (1000 Hz). B Water-suppressed spectrum from the same voxel demonstrates a fat peak around the typical position of 1000 Hz. Note the different scaling of the y axis. The fat fraction was calculated by dividing the amplitude of the fat peak by the water amplitude (Fig. A). MRS: magnetic resonance spectroscopy. [file 13395_2023_331_MOESM2_ESM.pptx]

## Slide 1
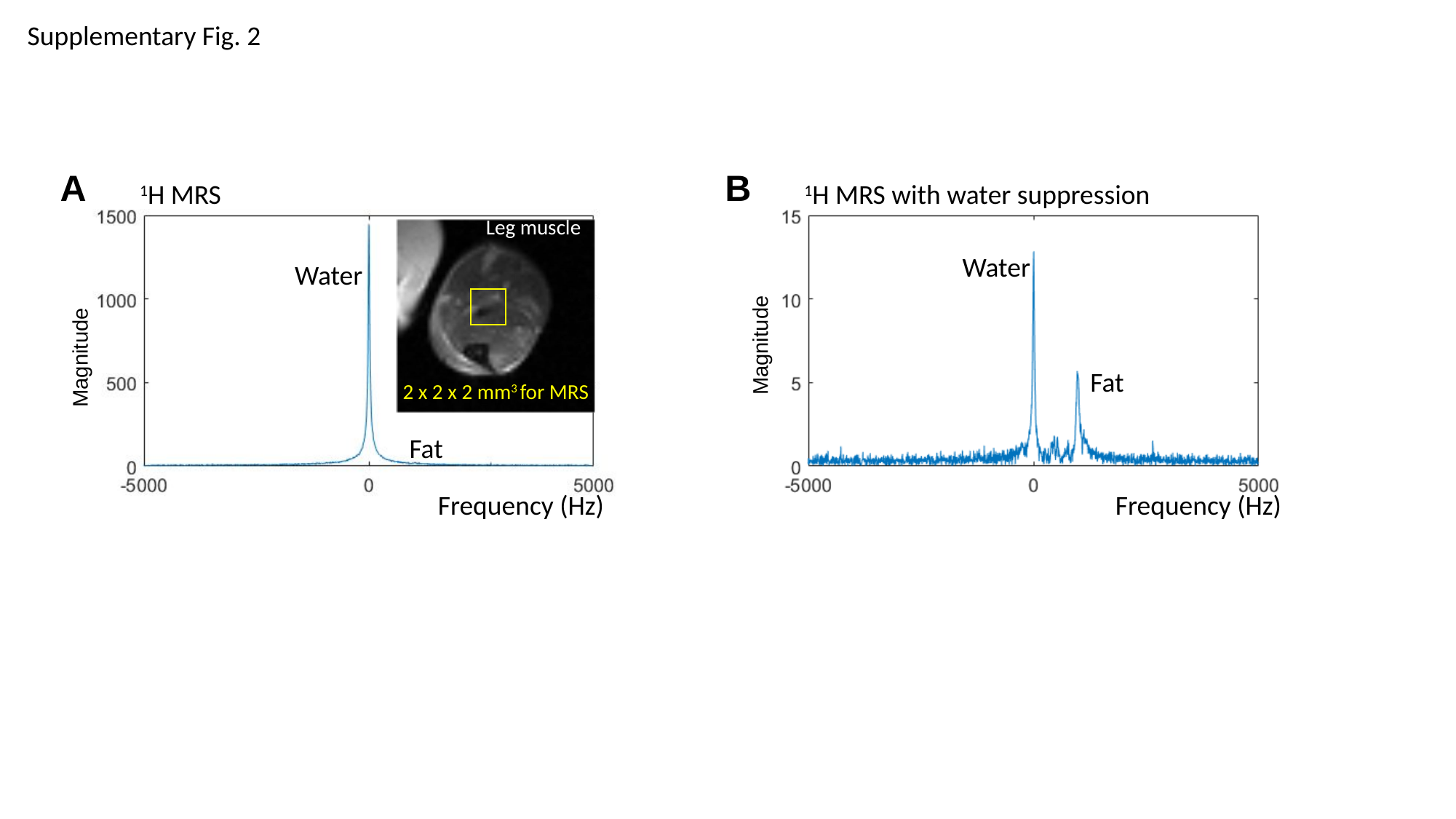

Supplementary Fig. 2
A
B
1H MRS
1H MRS with water suppression
Leg muscle
Water
Water
Magnitude
Magnitude
Fat
2 х 2 х 2 mm3 for MRS
Fat
Frequency (Hz)
Frequency (Hz)
